# New Principles of Polymer Composite Preparation. MQ Copolymers as an Active Molecular Filler for Polydimethylsiloxane Rubbers

**DOI:** 10.3390/polym13172848

**Published:** 2021-08-25

**Authors:** Ivan B. Meshkov, Aleksandra A. Kalinina, Vadim V. Gorodov, Artem V. Bakirov, Sergey V. Krasheninnikov, Sergei N. Chvalun, Aziz M. Muzafarov

**Affiliations:** 1 Enikolopov Institute of Synthetic Polymeric Materials Russian Academy of Sciences (ISPM RAS), Profsoyuznaya 70, 117393 Moscow, Russia; ivanbm@ispm.ru (I.B.M.); kalinina@ispm.ru (A.A.K.); gorodovvv@ispm.ru (V.V.G.); bakirov.artem@gmail.com (A.V.B.); derevatograf@yandex.ru (S.V.K.); s-chvalun@yandex.ru (S.N.C.); 2 National Research Center “Kurchatov Institute”, Akademika Kurchatova pl., 1, 123182 Moscow, Russia; 3 A.N. Nesmeyanov Institute of Organoelement Compounds, Russian Academy of Sciences, Vavilov St., 28, 119991 Moscow, Russia

**Keywords:** molecular filler, molecular composites, MQ copolymer, carboxyl-containing PDMS, mechanical properties, PDMS-based composition

## Abstract

Colorless transparent vulcanizates of silicone elastomers were prepared by mixing the components in a common solvent followed by solvent removal. We studied the correlation between the mechanical behavior of polydimethylsiloxane (PDMS)-rubber compositions prepared using MQ (mono-(M) and tetra-(Q) functional siloxane) copolymers with different ratios of M and Q parts as a molecular filler. The composition and molecular structure of the original rubber, MQ copolymers, and carboxyl-containing PDMS oligomers were also investigated. The simplicity of the preparation of the compositions, high strength and elongation at break, and their variability within a wide range allows us to consider silicone elastomers as a promising alternative to silicone materials prepared by traditional methods.

## 1. Introduction

The control of polymers and polymer compositions’ properties by regulating the structure of composition macromolecular components has been, and remains, the most important tool of chemists in their movement towards materials with superior properties [1,2]. Elastomeric materials based on polydimethylsiloxane (PDMS) rubbers are now widely used as rubbers, sealants, and coatings for various purposes [3,4]. The majority of the practical applications of PDMS elastomers are not feasible without silica fillers [5,6,7]. The role of this filler is perhaps not so important in any other elastomer as it is in PDMS. In many cases, the filled analogues demonstrate strength characteristics by a factor of 500 or more. The unique features of this effect are due to several reasons: first, the level of intermolecular interactions in PDMS is very low, so the initial characteristics are also low; second, the surface energy is also very low; and, finally, PDMS elastomers possess a unique affinity to silica fillers, especially aerosil, with a highly developed surface. Owing to a combination of these factors, the aerosil-filled PDMS raw rubber was actually a nanocomposite material long before this term had been introduced. The search for ways to enhance the strength of PDMS elastomers by applying efficient filling has continued. However, the subsequent increase in strength amounted to percent units or, at best, dozens of percent units rather than orders of magnitude, as was the case with the PDMS-aerosil composites. Nevertheless, new approaches to more efficient filling, pioneered by J.E. Mark [8,9,10,11,12,13,14,15,16,17], have been elaborated. J. E. Mark et al. [17] used standard methodologies as well in experiments with silica fillers with surfaces of various morphology. However, the elasticity modulus of nanocomposites filled with 50 wt.% of silica did not exceed 5.2 MPa, [12], while in the case of 20 wt.% filling it varied in a range from 1.01 to 2.21 MPa [16], depending on the preparation procedure and composition of the filler. Studies of liquid-phase filling have shown significant prospects for a further enhancement of mechanical characteristics [8,11,14]. The introduction of new chemically reactive oligomers that have catalytic activity, among other features, significantly boosted the studies in this area. The development of efficient catalysis opened preparation prospects for reactive filling [18,19]. However, some restrictions on the thickness of specimens persisted, which were related to the diffusion of the water vapors needed for hydrolytic polycondensation and the released alcohol.

The progress in studying new active molecular fillers rekindled the studies searching for new highly efficient fillers that can significantly simplify the production of filled elastomeric materials without deterioration of their operational characteristics and possibly even with their improvement. MQ copolymers [20] are promising candidates for this role.

The history of the practical application of MQ copolymers, or MQ resins as they are most frequently referred to, is very rich, though a realistic understanding of their structure (core—shell) has only formed rather recently [21]. The application of MQ resins is primarily related to pressure-sensitive adhesives [22], plasticizer additives [23], etc. [24,25,26]. The properties of these unique systems are known to be related to the ratio of the M and Q units. The MQ copolymers with an M:Q ratio of 1:1.5 are most widely used. The versatility of these systems was taken for granted for a long time, and only new discoveries of the composite nature of this complex polymer system enabled their studies to be brought to the standard “structure—properties” paradigm [20,27,28,29,30,31,32,33]. It was found that the properties of MQ copolymers are determined not only by the ratio of their units but also by the core—shell ratio in each individual macromolecule.

Thus, until recently, MQ copolymers have been used as additives to polymer compositions for purely empirical reasons—both as an additive and a component that improves the properties of the formulation as a whole. It is only after numerous trial and error, and utilization of nanogel fillers in various polymer systems [34,35,36,37,38], that it became clear that the MQ copolymers can be considered as a unique filler with a core—shell structure. The core in this system has much in common with other nanosized silica fillers, such as aerosils or white soot. An advantage of MQ systems compared with other silica fillers is their dual nature as macromolecules and particles. The molecular nature of filler allows one to regulate its dimensions and properties by modifying the structure, including the degree of affinity to the polymer matrix. For example, they do not require anti-texturing additives to be used in preparing PDMS-based rubber mixtures for preventing the “cold vulcanization” effects due to the formation of a network of hydrogen bonds between aerosil and raw rubber. Another important fact is that MQ resins may be easily dispersed in the PDMS matrix at the molecular level, while the extent of adsorption interactions may be controlled through the structural parameters of the MQ copolymers used, including the presence of the hydroxy groups within them. The latter feature enables MQ copolymers to act as linking agents, provided that the functional groups of raw rubber are sufficiently reactive toward the silanol functions of MQ copolymers. Amino and carboxy substituents can serve as such agents prone to interaction with silanols; owing to the ease of incorporating them into the PDMS structure, the use of these compounds is very promising [39,40,41,42,43].

The purpose of this study was to obtain elastic formulations based on functionalized liquid PDMS rubbers and organic solutions of MQ copolymers with various ratios of M and Q units, and to study their mechanical properties. This compound has vast combinatorial options to control the parameters of the polymer network and regulate the level of interactions between the macromolecules of different architecture.

## 2. Materials and Methods

The reagents and auxiliary materials used in the work were as follows: methyl tert-butyl ether (MTBE) reagent grade (EKOS-1, Moscow, Russia), 3-aminopropyltriethoxysilane 98% (ABCR, Karlsruhe, Germany), hydrochloric acid 0.1N analytical grade (Component reactive, Moscow, Russia), acetic acid reagent grade (EKOS-1, Moscow, Russia), anhydrous sodium sulfate reagent grade (EKOS-1, Moscow, Russia), tetraethoxysilane reagent grade (EKOS-1, Moscow, Russia), methyltrimethoxysilane 97% (ABCR, Karlsruhe, Germany), dimethylvinylchlorosilane 97% (ABCR, Karlsruhe, Germany), trimethylchlorosilane 97% (ABCR, Karlsruhe, Germany), pyridine reagent grade (EKOS-1, Moscow, Russia), toluene analytical grade (Component reactive, Moscow, Russia), α,ω-hydroxypolydimethylsiloxane «SKTN A» (SAZI, Moscow, Russia), and α,ω-hydroxypolydimethylsiloxane «SKTN E» (SAZI, Moscow, Russia). Pyridine was dried under molecular sieves 3Å, and the other above-listed substances were used as received.

We used the following materials to obtain new polydimethylsiloxane compounds:

-commercially available hydroxyl-terminated polydimethylsiloxanes of SKTN A and SKTN E brands with different molecular weights, modified with 3-aminopropyldiethoxysilyl groups at the chain ends: 

***P1*** based on «SKTN A», M_n_ = 18,500, M_w_ = 31,500, M_w_/M_n_ = 1.7 (Figures S1 and S6 in Supplementary Materials) containing 0.14 wt.% of amino groups;

***P2*** based on «SKTN E», M_n_ = 72,200, M_w_ = 120,000, M_w_/M_n_ = 1.7 (Figure S2 in Supplementary Materials) and 0.04 wt.% of amino groups;

-carboxyl-containing polydimethylsiloxane ***P3*** containing 6.8 mol.% of 12-carboxyundecyl groups distributed across the chain, Mn = 12,800, M_w_ = 21,600, M_w_/M_n_ = 1.7 synthesized using the technique reported in Ref. [44]; IR (cm^−1^): 3500-2500 (COO-H), 2990 (C-H), 1730 (C=O), 1445 (COO-H), 1400 (COO^−^), 1250 (CH_3_), 1075 (Si-O), 850 (Si-C); (Figures S3 and S6 in Supplementary Materials);

-methyl MQ resins that differ in the ratio of silica and trimethylsilyl groups:

***M2*** is an MQ copolymer [(CH_3_)_3_SiO_0,5_]_1_[SiO_2_]_2_ containing 5.8 wt.% OH groups, M_n_ = 10,700, M_w_ = 14,200, M_w_/M_n_ = 1.3; (Figure S4 in Supplementary Materials);

M2* is the ***M2*** resin that is additionally blocked with trimethylchlorosilane and does not contain residual hydroxy groups;

***M3*** is an MQ copolymer [(CH_3_)_3_SiO_0,5_]_1_[SiO_2_]_3_ containing 7.7 wt.% OH groups, M_n_ = 25,700, M_w_ = 224,200, M_w_/M_n_ = 8.7; (Figure S5 in Supplementary Materials).

**Figure 1:**
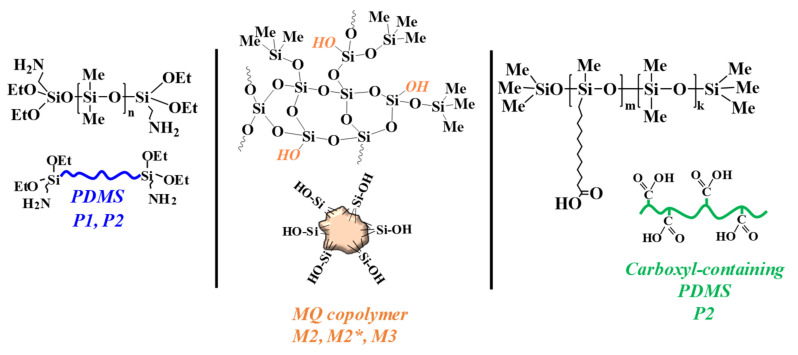
Formulas and schematic representations of the components used.

Figure 1 displays the structural formulas of the components used and their schematic representations.

***P1* synthesis.** A mixture of “SKTN A” brand PDMS (138 g, 0.006 mol) and 3-aminopropyltriethoxysilane (2.66 g, 0.012 mol) was stirred at 100 °C for 8 h, then vacuumed at 50 °C and 1 mm Hg to give 140 g of the product (yield 99%). The content of amino groups determined by titration (by 0.1N HCl) was 0.14 wt.%. ^1^H NMR (CDCl_3_, δ): 0.28–0.41 (m, 1106 H, (CH_3_)_2_Si), 0.55–0.67 (t, 2 H, CH_2_Si); 1.17–1.31 (t, 12 H, OCH_2_CH_3_); 1.46–1.66 (m, 2 H, CH_2_CH_2_Si); 2.64–2.75 (q, 2 H, CH_2_NH); 3.78–3.87 (q, 8 H, OCH_2_CH_3_).

***P2* synthesis. *P2*** was synthesized similarly to ***P1***. The content of amino groups determined by titration was 0.04 wt.%.

***M2* synthesis.** A mixture of tetraethoxysilane (207 g, 1 mol), methyltrimethoxysilane (51.8 g, 0.5 mol), and acetic acid (1074 g, 17.9 mol) was refluxed for 24 h at 125 °C. The products were washed with MTBE to a neutral pH of the aqueous layer and kept for 12 h above anhydrous sodium sulfate, then the solvent was distilled off to give 100 g of the product (yield 92%). ^1^H NMR (CDCl_3_, δ): 0.13 (m, 9 H, (CH_3_)_3_Si). IR (cm^−1^): 3500 (Si-OH), 2955 (C-H), 1250 (CH_3_), 1075 (Si-O), 850 (Si-C).

***M3* synthesis.** A mixture of tetraethoxysilane (234 g, 1.13 mol), methyltrimethoxysilane (39 g, 0.377 mol), and acetic acid (696 g, 11.6 mol) was refluxed for 24 h at 125 °C. The products were washed with MTBE to a neutral pH and kept for 12 h above anhydrous sodium sulfate, then the solvent was distilled off to give 118 g of the product (yield 95%). ^1^H NMR (CDCl_3_, δ): 0.13 (m, 9 H, (CH_3_)_3_Si). IR (cm^−1^): 3600 (Si-OH), 2960 (C-H), 1250 (CH_3_), 1080 (Si-O), 850 (Si-C).

The content of residual hydroxyl groups was analyzed by blocking the product with dimethylvinylchlorosilane under the conditions that did not violate the structure. The ratio of integral intensities of protons in vinyl and methylsilyl groups was determined similarly to the procedure reported in Ref. [45].

M2* synthesis. A solution of ***M2*** (10 g) in MTBE (150 mL) was added to a mixture of trimethylchlorosilane (32.6 g, 0.3 mol) and pyridine (23.2 g). The reaction mixture was refluxed for 4 h. The product was washed to a neutral pH of the aqueous layer and kept for 12 h above anhydrous sodium sulfate, then the solvent was distilled off to give 9.4 g of the product (yield 94 %). ^1^H NMR (CDCl_3_, δ): 0.13 (m, 9 H, (CH_3_)_3_Si). IR (cm^−1^): 2955 (C-H), 1250 (CH_3_), 1075 (Si-O), 850 (Si-C).

**^1^H Nuclear magnetic resonance (^1^H NMR)** spectra in solutions were acquired using the Avance AV-300 spectrometer (300 MHz) (Bruker, Bremen, Germany). CDCl_3_, having a chemical shift of δ = 7.25 ppm, was used as the internal standard and as a solvent.

**Gel permeation chromatography (GPC)** analysis was performed on a Shimadzu LC-10A series chromatograph (Shimadzu, Kioto, Japan), equipped with an RID-10A refractometer and SPD-M10A diode matrix detectors. Analytical separation was performed using a 7.8 mm × 300 mm Phenomenex column (USA) filled with Phenogel sorbent with a pore size of 15−500 Å. Toluene was used as the eluent. 

**IR spectra** were recorded on an IR Fourier spectrometer—Nicolet iS50 (Thermo Scientific, Waltham, MA, USA). 

**Procedure for the production of composites.** The required amounts of the components were dissolved in 5 mL of MTBE, stirred for 2–3 min, poured on a cellophane^®^ substrate, and kept for 3 days at room temperature. Ratio of the components varied as follows: for two-component mixtures 100 wt. parts of ***P1*** or ***P2*** per 0÷100 wt. parts of ***M2***, M2*, or ***M3***; for three-component mixtures 85 wt. parts of ***P1*** or ***P2*** and 15 wt. parts of ***P3*** per 0÷100 wt. parts of ***M2***, M2*, or ***M3***. The components were loaded on the basis of obtaining 1.44 g of vulcanizate with a thickness of 0.25 ± 0.02 mm and a diameter of 75 mm. A complete list of the molecular composites obtained and concentration of their components is presented in Table 1.

**Determination of the gel fraction in the composites**. The composites weighed with high accuracy (0.0001 g) were placed into a Soxhlet apparatus equipped with a reflux condenser and were treated with a boiling solvent (toluene). The extraction was carried out over 15 h. The calculations were performed by the formula: gel fraction = m_1_*100/m_0_, where m_0_ and m_1_ are the masses of the material sample before and after the extraction, g.

**The mechanical properties** were studied using an Instron-5965 versatile test device equipped with a load sensor for ± 50 N in the uniaxial single tension mode and in the cycling mode. Instrumental error was as follows: when measuring the load, ± 0.5% of the measured value; when measuring the displacement, ± 0.05% of the full scale or ± 0.5% of the measured value (whichever is greater). The shape of the test samples was in accordance with ASTM 638 (Type 5). The specimen thickness was 0.25 ± 0.02 mm. The initial deformation rate was 60 mm/min. Prior to testing, the specimens were conditioned in air at 23 °C for 24 h. Specific mechanical characteristics (elasticity modulus and ultimate strength) were calculated using the measured linear dimensions. The cycling limits were 5–50% of the fracture strain. The number of cycles in the cyclic tests was 100, after which testing was continued until rupture. The properties of all produced molecular composites were studied prior to and after heat treatment (HT) at 200 °C for 2 h. Three samples for each composition were investigated. For a statistical assessment of the data obtained, standard deviations were calculated for all basic constants (strength, elastic modulus, fracture strain).

## 3. Results

### 3.1. Preparation of Elastomeric Composites 

A wide range of formulations, differing in concentrations, components, and content of the functional groups within them, were obtained by adding methyl MQ resins with residual hydroxysilyl groups and carboxyl-containing PDMS to commercially available PDMS terminated with 3-aminopropyltriethoxysilane (***P1*** and ***P2***).

The amount of carboxyl-containing PDMS was 15 wt. parts per 85 wt. parts of ***P1*** or ***P2***. The amount of MQ was varied within a range from 0 to 100 wt. parts per 100 wt. parts of the total PDMS in the formulation. Films were obtained by pouring a solution containing the required amounts of the components in MTBE onto a cellophane® substrate. All MQ-based formulations, regardless of the variation in the composition, rapidly solidified at room temperature to form transparent elastomeric materials. The formation of dense films is observed as early as 5–30 min after the components are mixed, depending on the composition. Heat treatment of the samples was performed for 2 h at 200 °C. The yield of the composite gel fraction was, in all cases, higher than 90% (with the exception of Sample 2, containing a mixture of blocked and unblocked MQ resin), following the results of the extraction of the films with toluene in a Soxhlet extractor before and after HT (Table 1). This result demonstrates the high efficiency of the curing procedure both before and after HT.

Changing the PDMS-MQ composites’ filler content allows for the control of the mechanical behavior of the obtained samples.

### 3.2. Mechanical Characteristics of the Elastomer Composites

**Figure 2:**
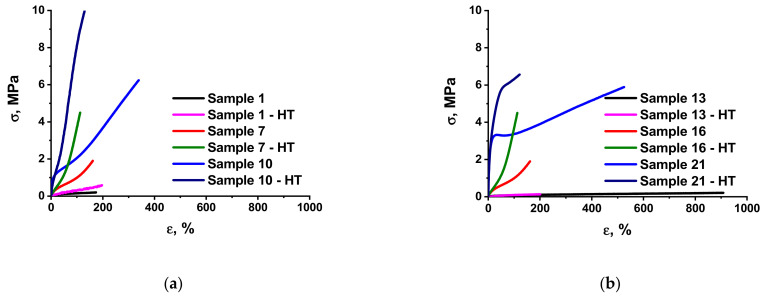

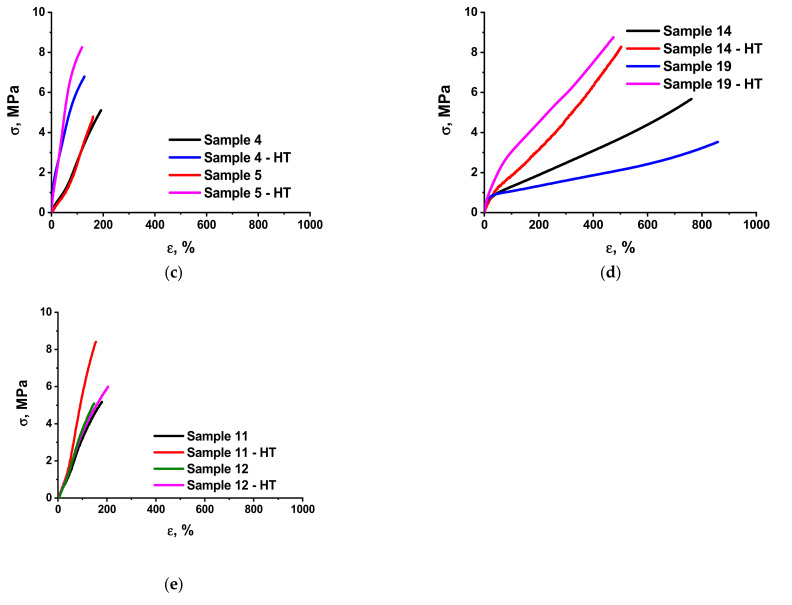
Deformation curves of molecular composites before and after HT: (**a**) Composites based on ***P1*** containing ***P3***, filled with 16,67 wt.% (Sample 7) or 33,33 wt.% (Sample 10) ***M2*** and without (Sample 1) ***M2***; (**b**) Composites based on ***P2*** containing ***P3***, filled with 16,67 wt.% (Sample 16) or 50,00 wt.% (Sample 21) ***M2*** and without (Sample 13) ***M2***; (**c**) Composites based on ***P1*** not containing ***P3***, filled with 16,67 wt.% ( Sample 4) or 33,33 wt.% (Sample 2) ***M2***; (**d**) Composites based on ***P2*** not containing (Sample 14) or containing (Sample 19) ***P3***, filled with 33,33 wt.% ***M2***; (**e**) Composites based on ***P1*** not containing (Sample 11) or containing (Sample 12) ***P3***, filled with 16,67 wt.% ***M3***.

Analysis of the mechanical behavior of the molecular composites has shown that, by varying their composition, ratio of components, and their functionality, one can control the strength properties of the materials within a very wide range. Figure 2 shows examples of typical deformation curves for the materials obtained, based on either PDMS ***P1*** or ***P2***.

The main mechanical characteristics of the molecular composites based on ***P1*** and ***P2*** are presented in Table 2 and Table 3, respectively. The presented plots (Figure 2a–e) show the effect of each component and HT on the mechanical behavior of the systems in question.

The effect of the molecular wt. of liquid PDMS rubber may be assessed by comparing the relative elongations of specimens (Table 2 and Table 3), defining the distance between the junctions—the main parameter of the future network. While this quantity does not exceed 300% for the ***P1*** raw rubber, values of 500 or more can be reached for ***P2***.

While raw rubber that sets the cross-linked distances of the main network is the basis of the formulation, an MQ resin is a tool to control the properties. The effect of the MQ copolymer can be reduced to a minimum. If MQ with preliminarily and carefully blocked groups is used, it performs as an inert filler, and its effect on the formulation properties is not large (Table 2, Sample 2). However, the data in Table 2 and Table 3 clearly show how a gradual increase in the fraction of hydroxyl-containing MQ copolymer ***M2*** in the formulation results in a significant improvement of the mechanical behavior of elastomer composites based on ***P1*** (Table 2, Samples 3–5 and 6–10) or ***P2*** (Table 3, Samples 15–21). The molecular parameters of MQ are also of importance. A transition from ***M2*** to ***M3***, i.e., to systems with a less shielded silica core, results in an increase in elasticity modulus, tensile strength, and relative elongation in a series of formulations of the same type (Table 2, Samples 4 and 11, 7 and 12).

**Figure 3:**
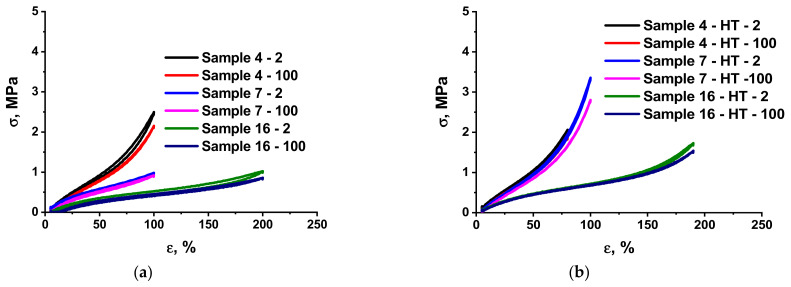

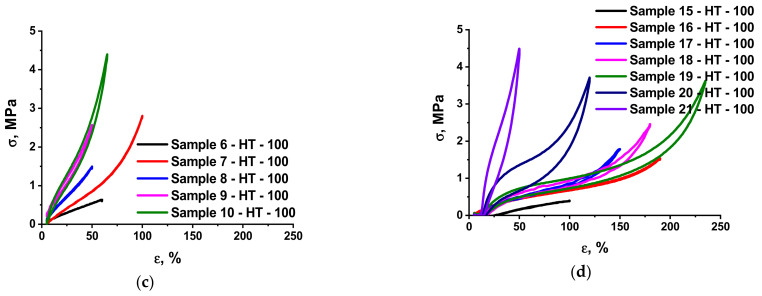
Dynamics of changes in mechanical hysteresis (2nd and 100th cycles) in composites with various compositions before and after HT: (**a**) Corresponds to the composites filled with 16.67 wt.% ***M2*** based on ***P1*** (not containing (Sample 4) or containing (Sample 7) ***P3***) and ***P2*** (containing (Sample 14) ***P3***) before HT; (**b**) Corresponds to the composites filled with 16.67 wt.% ***M2*** based on ***P1*** (not containing (Sample 4) or containing (Sample 7) ***P3***) and ***P2*** (containing (Sample 16) ***P3***) after HT; (**c**) Corresponds to the thermally treated composites based on ***P1*** and ***P3*** containing 9.09 to 33.33 wt.% ***M2*** (100th cycles) (Samples 6–10); (**d**) Corresponds to the thermally treated composites based on ***P2*** and ***P3***, containing 9.09 to 50.00 wt.% of ***M2*** (100th cycles) (Samples 15–21).

Estimating the energy dissipation level in reversible deformation cycles is crucial to describe the properties of elastomer composites. Polysiloxane elastomers are especially convenient for this purpose, owing to low energy losses in conformation transitions [46]. Analysis of cyclic tests of the molecular composites obtained (Figure 3 and Table 4) has shown that the addition of an active filler, an MQ copolymer, is accompanied by the expected growth in the hysteresis loop. 

To obtain a complete picture of the effect of composition and heat treatment on mechanical hysteresis, we present the results for losses in the second and hundredth load–unload cycles. This is important since, as can be seen from Table 4, regardless of the composition, the hysteresis in the first cycles is noticeably greater than in the hundredth. Such comparatively high energy dissipation in the first cycles can be associated with the structural reorganization (ordering and network optimization) and conformational changes. The degree of ordering depends on the lability of the individual components of the system. Thus, the difference in energy dissipation at the first and subsequent stages of cycling should depend on the composition and heat treatment, and also needs to be analyzed.

Losses increased significantly, which is especially pronounced in the case of a filling above 16 wt.%, both in the first and subsequent stretching-contraction cycles. For example, if the filling degree of the initial ***P1***-based composites increased from 16 to 33 wt.%, the hysteresis loop area increased from 2 to 14% after 100 stretching cycles (Table 4, Samples 4 and 14, Figure 3A). Such significant energy losses are caused by matrix flow due to the destruction of weak interactions with the filler. An efficient technique to reduce the contribution of this phenomenon to the overall energy losses involves the HT of the composite accompanied by the formation of strong covalent bonds between the matrix and the MQ resin, which should prevent plastic flow. Indeed, cyclic tests have shown that HT of the molecular composites with all compositions prepared significantly reduces losses. For example, the hysteresis loop of the ***P1***-based composite containing 16 and 33 wt.% of MQ copolymer decreased to 1 and 10%, respectively, after HT (Table 4, Samples 4 and 14, respectively, Figure 3B). 

On the addition of ***P3*** PDMS that contains carboxy groups distributed along the chain to the system, hysteresis increased to 6 and 30% (for 16 and 33 wt.% filling, respectively) but decreased significantly to 1 and 16% after HT (Table 4, Samples 6 and 19, respectively). Such changes can be explained in terms of transformations of the hydrogen bond network, which will be discussed below, due to addition of carboxyl-containing oligomers.

Increasing the fraction of the silica core in the MQ copolymer on transition from ***M2*** to ***M3*** resulted in a twofold increase in hysteresis (Table 4, Samples 4 and 11, 6 and 12, respectively, Figure 3A,B). This difference persisted after the HT. Such an enhancement of hysteresis phenomena may be explained by an increase in the number of labile hydrogen bonds caused by an increase in the concentration of hydroxy groups, which are not involved in the formation of the main chemical network in the structure of the MQ copolymer, in the case of ***M3***.

The studies we performed have shown that, in the case of moderate filling levels of 16–23 wt.% (Table 4, Samples 3, 4, 5, 6, 8, 10, 11, 12; Figure 3C,D), the thermally treated molecular composites based on low-molecular PDMS feature an advantageous combination of strength indicators and low energy losses in alternating-load cycles of 1–4 % (100 load–unload cycles), owing to which they can be considered as a basis for many practical applications.

HT of the composites studied is not mandatory since vulcanized rubber preserves its shape and loses stickiness rather rapidly even without HT. However, a comparison of specimens that underwent HT with their cold-vulcanization predecessors shows high-temperature annealing results in the completion of all chemical reactions in a specimen, and ensures that its characteristics are stable over time.

## 4. Discussion

**Figure 4:**
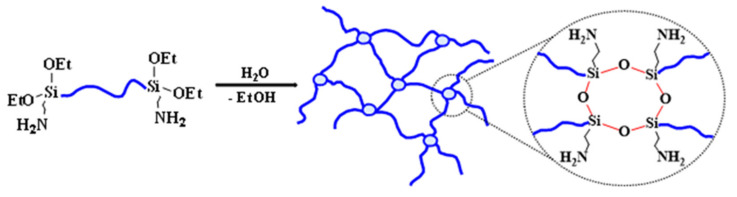
Curing scheme for 3-aminopropyl diethoxysilyl-terminated PDMS (***P1*** or ***P2***).

Despite the “apparent” simplicity and speed of the procedure of rubber vulcanization, it is actually a complex multilevel process of competing interactions that involves chemical (formation of chemical bonds) and physical (hydrogen bonds and other polar interactions) aspects. The complexity of the process is due to the variety of functional groups and, as mentioned above, the variety of possible reactions. To obtain a general understanding of the network structure being formed, we will proceed stepwise, and identify those steps which are the most important in the effect they have on the properties of the elastomers formed. Let us start with the observation that raw rubber with terminal 3-aminopropyl(diethoxy)silyl groups is self-sufficient and can form its own network upon hydrolysis of ethoxy groups, and homo- and heterofunctional condensation of the silanols produced, catalyzed by amino groups proactively delivered in advance to the place of the reaction. The material that is formed is a typical unfilled siloxane formulation with a low elasticity module and high elongation (Figure 4). It should be mentioned that, in the last case, the curing process took much longer (weeks) which has not been accelerated, even by HT. 

The application of these materials is very limited; they can be obtained without a preliminary modification of raw rubber [5,47].

**Figure 5:**
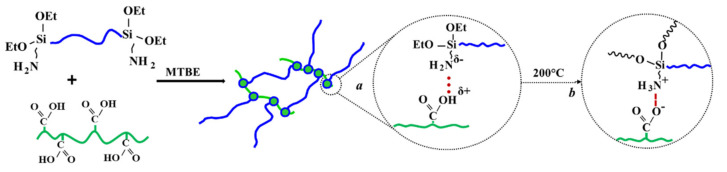
Curing scheme for two-component formulations based on 3-aminopropyl diethoxysilyl-terminated and carboxyl-containing PDMS.

Adding a carboxyl-containing component to the formulations makes it possible to introduce a second component into the network structure by the reaction of 3-aminopropyl groups with carboxy groups. A second component, thus, appears in the network structure, which is bound to the first one but has different network parameters. The second network is formed at room temperature by weak hydrogen bonds (Figure 5, a); therefore, the elongation at break in ***PE***-based formulations is as large as 900%. However, they strengthen significantly after the treatment, and seem to pass into chemical bonds (Figure 5, b). The elongation at break is then determined by the second network, and decreases to 198% (Table 3, Sample 13). 

**Figure 6:**
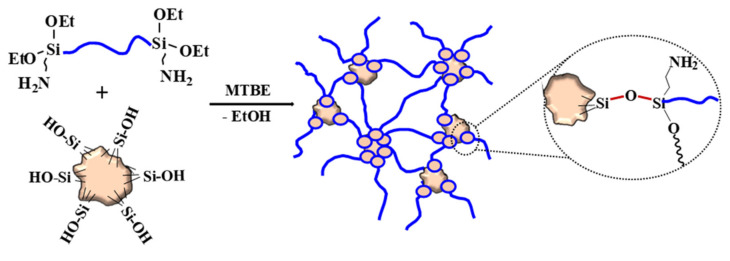
Curing scheme for two-component formulations based on 3-aminopropyl diethoxysilyl-terminated PDMS and MQ copolymer.

Adding small amounts of the MQ copolymers that contain residual hydroxy groups to raw rubber enables the formation of a network even without traces of moisture. The process occurs in heterofunctional condensation mode, and network nodes become multicentered clusters (Figure 6).

The addition of an MQ copolymer with preliminarily blocked silanol groups to the formulation does not significantly affect the properties of vulcanized rubber (Table 2, Sample 2). However, an increase in the concentration of a hydroxyl-containing MQ copolymer in a two-component formulation results in a significant enhancement of the mechanical properties of the formulation. This effect may be entirely attributed to the adsorption interactions between the MQ copolymer and raw rubber chains, i.e., the aerosil effect that determines the properties of siloxane elastomers, as we emphasized above. 

Carboxyl-containing oligomers ***P3*** were used as an auxiliary additive. They do not have a significant effect on the formulation properties, but can speed up the formulation “hardening” immediately after mixing, and neutralize the PDMS amino groups during HT. One cannot rule out that their structure is rearranged during HT, which has been well observed if carboxyl-containing PDMS are used as the formulation basis [48,49]. However, this effect is insignificant compared to the reaction between PDMS in raw rubber and an MQ copolymer, and may probably be enhanced by more thorough tuning of the concentration of carboxy groups, the amount of the components added, and the additional treatment of carboxy groups already contained in a formulation.

## 5. Conclusions

This study presents a new approach to the design of elastomeric materials based on MQ copolymers. This technology exploits the dualistic properties of these objects that have both properties of macromolecules and colloid particles. MQ copolymers exhibit their molecular nature as homogeneous linking agents in the presented composites. They are also very efficient as active fillers. These fillers significantly enhance the mechanical indicators of vulcanized rubber: they are not inferior to aerosils but do not require a heterophase mixing of ingredients. Finally, they are efficient in the blocked version, and as inert fillers that slightly enhance the composition strength and elongation at rupture.

The data reported in the study show that the potential of MQ systems as a versatile ingredient is far from being solved. The ranges of its molecular parameters that may be changed very finely were only roughly determined. This also refers to the degree of MQ dispersion by molecular wt. and size, which are the same in this case, and the control of the concentration of hydroxy groups. This study clearly shows that these groups are important in regulating the properties of vulcanized rubbers. 

The role of carboxyl-containing modifiers has only been explored as an additional topic. The addition of these substances only speeds up cold vulcanization, which was not the focus of our study. Nevertheless, the reported data clearly show that these additives affect the properties of vulcanized rubber to some extent. This implies that the reasonable control of its molecular parameters and optimization of concentrations also deserve detailed studies. 

Perhaps, exploration of the morphology and phase behavior of the vulcanized rubber obtained would be an extremely important continuation of this study. What will be the final state that a molecular solution of the initial formulation will acquire upon vulcanization and subsequent HT? This issue, which requires a detailed study and understanding, is the most urgent in our further work.

## Figures and Tables

**Table 1:** Compositions of molecular composites obtained.

Sample	Composite’s Abbreviation *^a^*	Components Content in Composites, wt.%					Gel Fraction *^b^*, wt.%
		*P1*	*P2*	M2 (M2*)	*M3*	*P3*	
1	P1/85-P3/15	85.00	-	-	-	15.00	96.6
2	P1/100-M2/3.5-M2*/50	65.15	-	2.12 (32.57)		-	79.2/68.8 *^c^*
3	P1/100-M2/3.5	96.62	-	3.38	-	-	92.3/85.8 *^c^*
4	P1/100-M2/20	83.33	-	16.67	-	-	99.1/95.4 *^c^*
5	P1/100-M2/50	66.67	-	33.33	-	-	98.7
6	P1/85-M2/10-P3/15	77.27	-	9.09	-	13.64	99.5
7	P1/85-M2/20-P3/15	70.83	-	16.67	-	12.50	97.9/94.1 *^c^*
8	P1/85-M2/30-P3/15	65.38	-	23.08	-	11.54	99.5
9	P1/85-M2/40-P3/15	60.72	-	28.57	-	10.71	99.4
10	P1/85-M2/50-P3/15	56.67	-	33.33	-	10.00	100
11	P1/100-M3/20	83.33	-	-	16.67	-	99.4
12	P1/85-M3/20-P3/15	70.83	-	-	16.67	12.50	95.7
13	P2/85-P3/15	-	85.00	-	-	15.00	74.4/5.8 *^c^*
14	P2/100-M2/50	-	66.67	33.33	-	-	99.2/95.4 *^c^*
15	P2/85-M2/10-P3/15	-	77.27	9.09	-	13.64	100/77.4 *^c^*
16	P2/85-M2/20-P3/15	-	70.83	16.67	-	12.5	98.2
17	P2/85-M2/30-P3/15	-	65.38	23.08	-	11.54	100
18	P2/85-M2/40-P3/15	-	60.72	28.57	-	10.71	97.4
19	P2/85-M2/50-P3/15	-	56.67	33.33	-	10.00	98.6
20	P2/85-M2/75-P3/15	-	48.57	42.86	-	8.57	96.9
21	P2/85-M2/100-P3/15	-	42.50	50.00	-	7.50	98.0

*^a^* Composite’s abbreviation: PDMS abbreviation (***P1*** or ***P2***)/ wt. parts of this component; MQ copolymer abbreviation (***M2***, M2* or ***M3***)/wt. parts of this component; carboxyl-containing PDMS abbreviation (***P3***)/wt. parts of this component if available. *^b^* the content of the gel fraction in vulcanized rubber. *^c^* the content of the gel fraction in vulcanized rubber before heat treatment.

**Table 2:** Mechanical properties of ***P1***-based composites before and after HT.

Sample	Composite’s Abbreviation	σ_max_, MPa		ε_b_, %		E, MPa	
		before	after	before	after	before	after
1	P1/85-P3/15	0.2 ± 0.01	0.6 ± 0.04	174 ± 14	197 ± 21	0.3 ± 0.01	0.4 ± 0.01
2	P1/100-M2/3.5-M2*/50	0.9 ± 0.1	1.6 ± 0.1	257 ± 16	331 ± 10	0.5 ± 0.05	0.5 ± 0.05
3	P1/100-M2/3.5	0.8 ± 0.1	0.9 ± 0.1	229 ± 11	229 ± 11	0.5 ± 0.04	0.6 ± 0.05
4	P1/100-M2/20	5.1 ± 0.1	4.8 ± 0.1	192 ± 21	162 ± 11	2.8 ± 0.1	2.0 ± 0.1
5	P1/100-M2/50	6.8 ± 0.3	8.2 ± 0.4	127 ± 11	118 ± 9	25.7 ± 0.2	16.3 ± 0.3
6	P1/85-M2/10-P3/15	0.9 ± 0.1	2.0 ± 0.1	112 ± 8	121 ± 8	1.2 ± 0.05	1.1 ± 0.04
7	P1/85-M2/20-P3/15	2.5 ± 0.1	4.5 ± 0.1	194 ± 16	113 ± 7	2.5 ± 0.1	2.4 ± 0.1
8	P1/85-M2/30-P3/15	4.6 ± 0.2	4.5 ± 0.1	193 ± 12	96 ± 12	6.5 ± 0.2	3.1 ± 0.1
9	P1/85-M2/40-P3/15	4.9 ± 0.2	7.0 ± 0.3	194 ± 18	101 ± 9	12.8 ± 0.2	9.1 ± 0.2
10	P1/85-M2/50-P3/15	6.2 ± 0.2	10.0 ± 0.4	339 ± 24	132 ± 11	16.0 ± 0.4	10.8 ± 0.2
11	P1/100-M3/20	5.2 ± 0.2	8.4 ± 0.3	179 ± 10	155 ± 10	3.2 ± 0.1	3.7 ± 0.1
12	P1/85-M3/20-P3/15	6.0 ± 0.3	5.1 ± 0.2	205 ± 13	148 ± 9	3.5 ± 0.1	3.3 ± 0.1

**Table 3:** Mechanical properties of ***P2***-based composites before and after HT.

Sample	Composite’s Abbreviation	σ_max_, MPa		ε_b_, %		E, MPa	
		before	after	before	after	before	after
13	P2/85-P3/15	0.2 ± 0.01	0.1 ± 0.01	900 ± 51	198 ± 12	0.1 ± 0.01	0.1 ± 0.01
14	P2/100-M2/50	5.7 ± 0.2	8.3 ± 0.3	761 ± 44	503 ± 24	3.8 ± 0.2	3.4 ± 0.2
15	P2/85-M2/10-P3/15	2.4 ± 0.2	1.8 ± 0.1	582 ± 32	302 ± 12	0.7 ± 0.1	0.7 ± 0.1
16	P2/85-M2/20-P3/15	1.9 ± 0.1	4.2 ± 0.2	393 ± 19	378 ± 20	1.7 ± 0.1	1.3 ± 0.1
17	P2/85-M2/30-P3/15	2.9 ± 0.2	5.8 ± 0.3	498 ± 22	437 ± 14	2.3 ± 0.1	2.2 ± 0.1
18	P2/85-M2/40-P3/15	3.1 ± 0.2	8.0 ± 0.3	560 ± 24	539 ± 22	5.2 ± 0.2	5.1 ± 0.2
19	P2/85-M2/50-P3/15	3.5 ± 0.2	8.6 ± 0.2	858 ± 39	453 ± 20	7.0 ± 0.2	6.5 ± 0.2
20	P2/85-M2/75-P3/15	5.7 ± 0.2	7.2 ± 0.3	575 ± 15	240 ± 11	24.4 ± 0.9	24.0 ± 0.7
21	P2/85-M2/100-P3/15	5.9 ± 0.3	6.6 ± 0.2	525 ± 19	121 ± 9	51.0 ± 2.1	47.3 ± 1.6

**Table 4:** Dynamics of changes in mechanical hysteresis before and after HT in the specimens studied.

Sample	Composite’s Abbreviation	MQ Content ^a^, wt.%	Hysteresis, %			
			before		after	
			2nd Cycle	100th Cycle	2nd Cycle	100th Cycle
4	P1/100-M2/20	16.67	7.12	2.10	3.93	1.21
6	P1/85-M2/20-P3/15	16.67	7.26	5.62	3.49	0.97
11	P1/100-M3/20	16.67	9.61	3.49	6.73	2.83
12	P1/85-M3/20-P3/15	16.67	16.72	5.22	6.95	2.42
8	P1/85-M2/30-P3/15	23.08	17.07	13.19	4.32	3.34
5	P1/100-M2/50	33.33	17.80	14.27	13.49	10.13
10	P1/85-M2/50-P3/15	33.33	39.72	29.79	17.67	15.71
14	P2/100-M2/50	33.33	29.78	17.43	22.09	12.64
19	P2/85-M2/50-P3/15	33.33	35.50	22.99	27.68	19.46
16	P2/85-M2/20-P3/15	16.67	13.01	8.48	4.29	2.46
2	P1/100-M2/3.5-M2*/50	2.12/32.57 ^b^	5.12	4.45	3.91	3.15
3	P1/100-M2/3.5	3.38	2.05	0.91	1.59	0.94

^a^ MQ content in the composition; ^b^ the content of M2*.

## Supplementary Materials

The following are available online at https://www.mdpi.com/article/10.3390/polym13172848/s1, Figure S1: GPC curve of ***P1***, Figure S2: GPC curve of ***P2***, Figure S3: GPC curve of ***P3***, Figure S4: GPC curve of ***M2*** and ***M2****, Figure S5: GPC curve of ***M3***, Figure S5: IR spectra of ***P3*** and composites before and after heating.
