# Supplementary material for: New Principles of Polymer Composite Preparation. MQ Copolymers as an Active Molecular Filler for Polydimethylsiloxane Rubbers
Source: Polymers (Basel). 2021 Aug 25;13(17):2848. doi: 10.3390/polym13172848 (PMC8433927; doi:10.3390/polym13172848)
Supplement: Supplementary file 1 [file polymers-13-02848-s001.zip › polymers-1346530-supplementary.pdf]

polymers

MDPI

Supplementary Materials: New Principles of Polymer Composite Preparation. MQ Copolymers as an Active Molecular Filler for Polydimethylsiloxane Rubbers

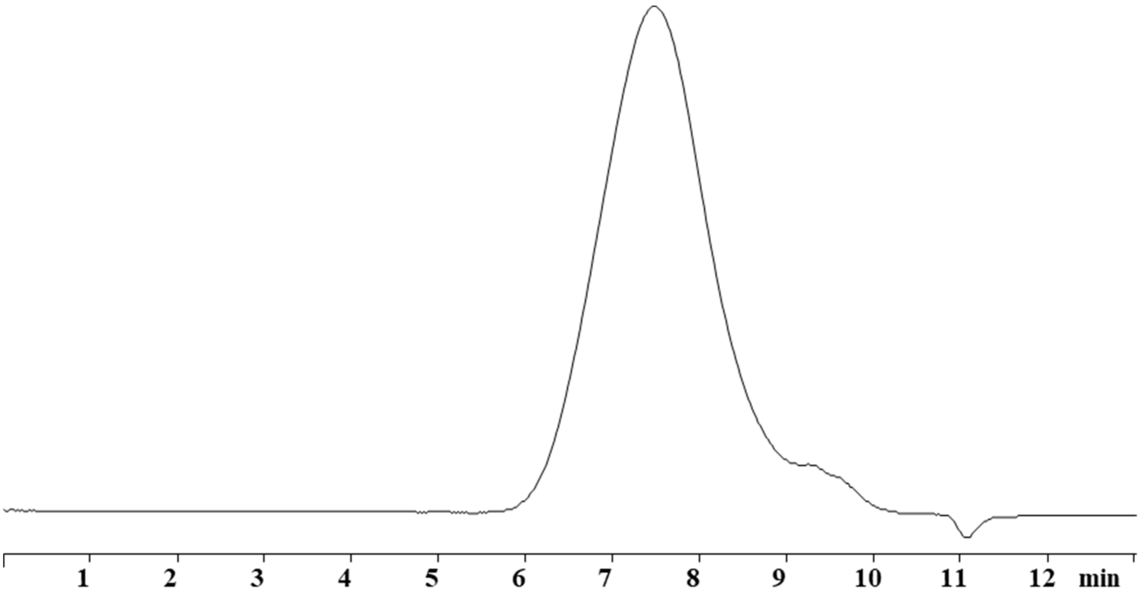

Figure S1. GPC curve of P1.

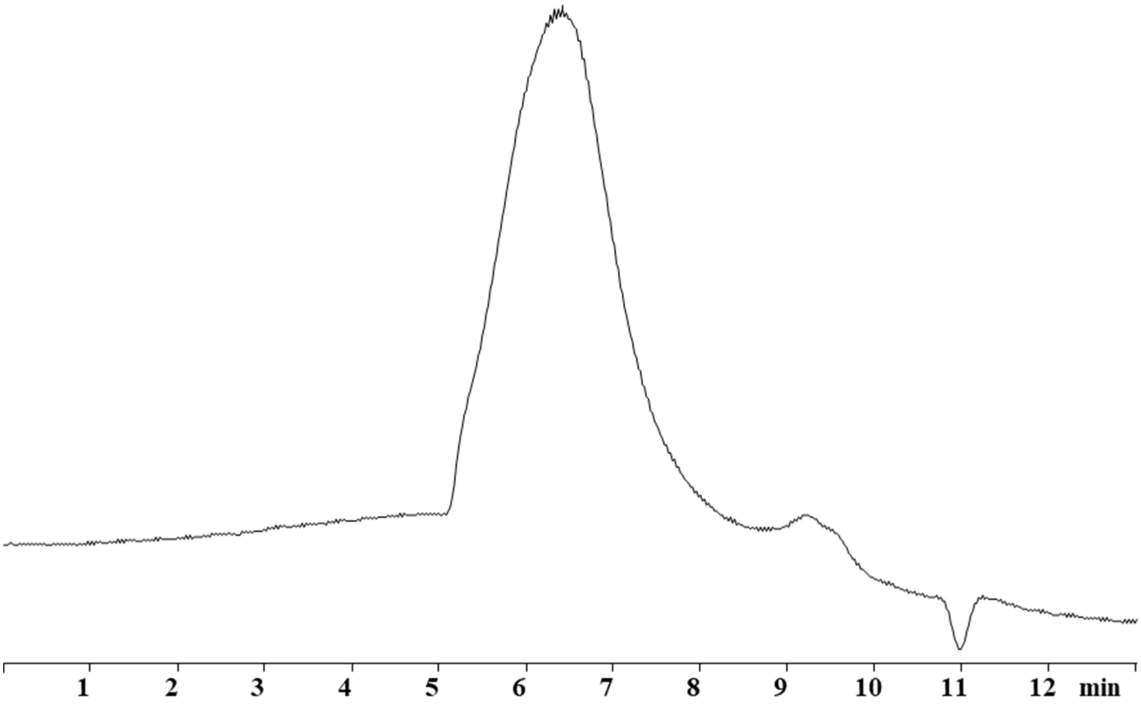

Figure S2. GPC curve of P2.

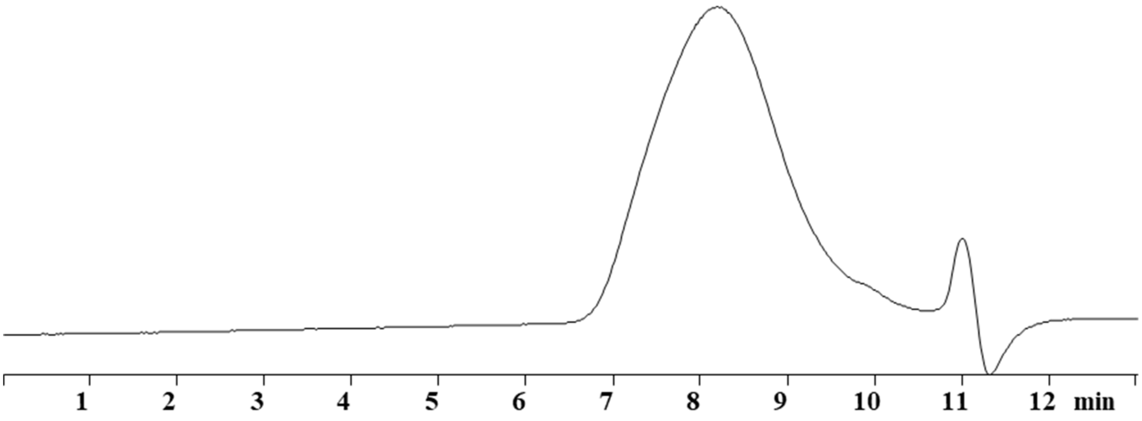

Figure S3. GPC curve of P3.

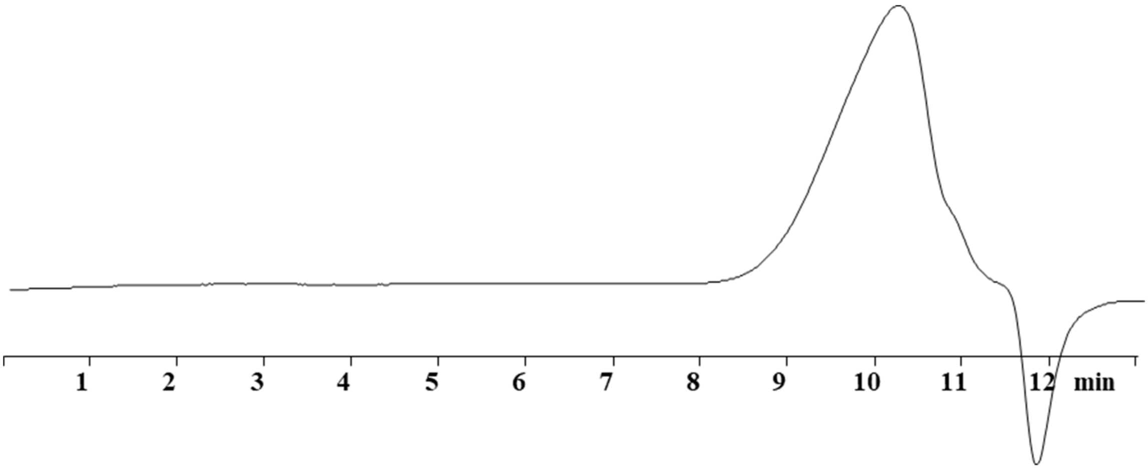

Figure S4. GPC curve of M2 and M2*.

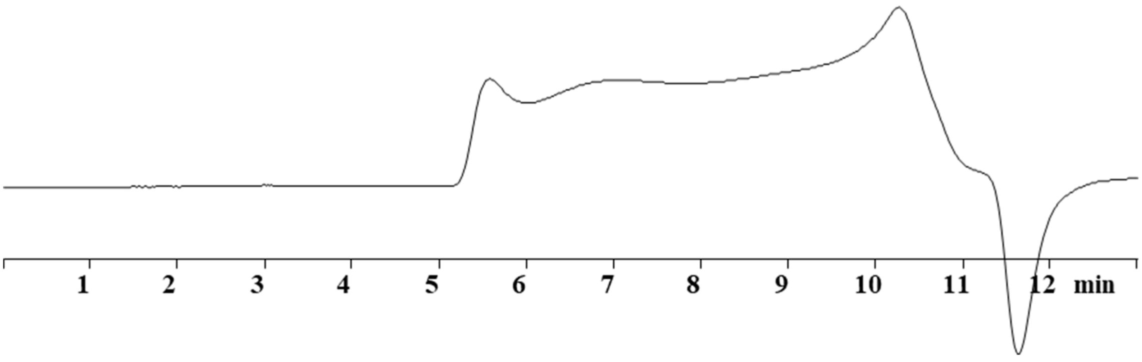

Figure S5. GPC curve of M3.

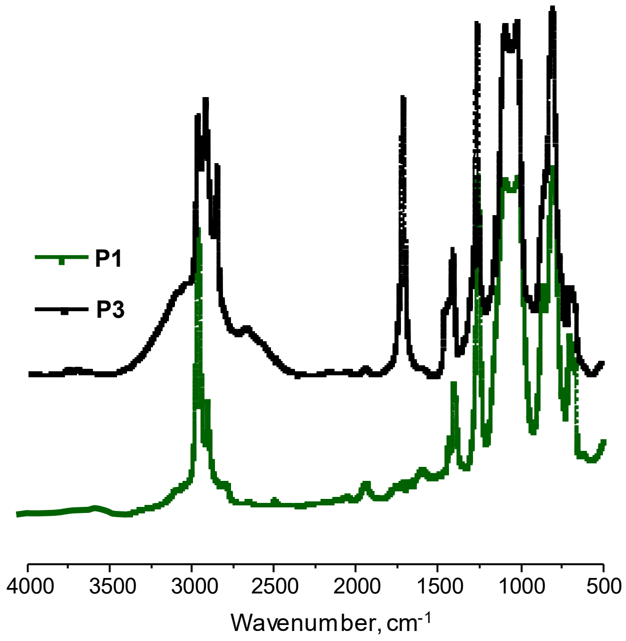

Figure S6. IR spectra of ***P1*** and ***P3***.

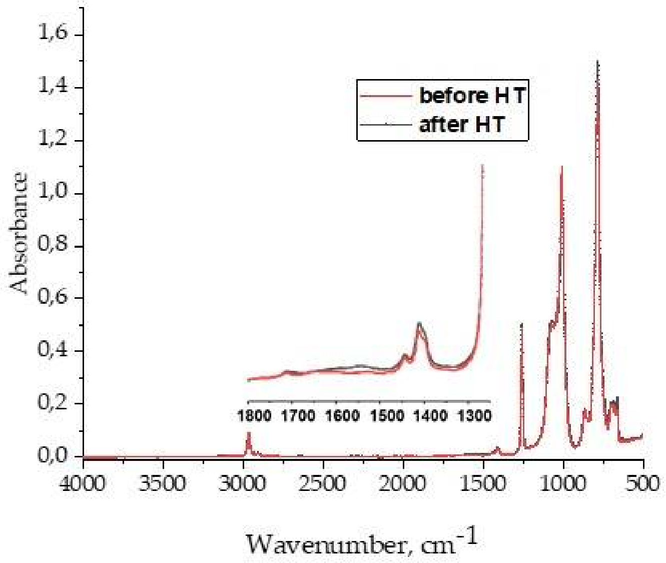

Figure S7. IR spectra of composite Sample 10 before and after heating.
